# Unlocking the role of non-coding RNAs in prostate cancer progression: exploring the interplay with the Wnt signaling pathway

**DOI:** 10.3389/fphar.2023.1269233

**Published:** 2023-09-27

**Authors:** Tong Bu, Le Li, Jiyu Tian

**Affiliations:** Department of Gastroenterology, The Fourth Affiliated Hospital of China Medical University, Shenyang, Liaoning, China

**Keywords:** non-coding RNA, miRNA, lncRNA, circRNA, Wnt signaling pathway, prostate cancer

## Abstract

Prostate cancer (PCa) is one of the most common cancers in males, exhibiting a wide spectrum of clinical manifestations that pose challenges in its diagnosis and treatment. The Wnt signaling pathway, a conserved and complex pathway, is crucial for embryonic development, tissue homeostasis, and various physiological processes. Apart from the classical Wnt/β-catenin signaling pathway, there exist multiple non-classical Wnt signaling pathways, including the Wnt/PCP and Wnt/Ca^2+^ pathways. Non-coding RNAs (ncRNAs) are involved in the occurrence and development of PCa and the response to PCa treatment. ncRNAs are known to execute diverse regulatory roles in cellular processes, despite their inability to encode proteins. Among them, microRNAs, long non-coding RNAs, and circular RNAs play key roles in the regulation of the Wnt signaling pathway in PCa. Aberrant expression of these ncRNAs and dysregulation of the Wnt signaling pathway are one of the causes of cell proliferation, apoptosis, invasion, migration, and angiogenesis in PCa. Moreover, these ncRNAs affect the characteristics of PCa cells and hold promise as diagnostic and prognostic biomarkers. Herein, we summarize the role of ncRNAs in the regulation of the Wnt signaling pathway during the development of PCa. Additionally, we present an overview of the current progress in research on the correlation between these molecules and clinical features of the disease to provide novel insights and strategies for the treatment of PCa.

## 1 Introduction

In males, prostate cancer (PCa) is one of the most common cancers and the fifth leading cause of cancer-related deaths (Sung et al., 2021). According to estimates for 2020, the worldwide incidence of PCa was 1.4 million new cases, with more than 375,000 males dying owing to the disease (Sandhu et al., 2021). PCa is a complex and heterogeneous disease, exhibiting a wide range of clinical manifestations, ranging from indolent to aggressive (He et al., 2022). Thus, investigating the mechanisms of the occurrence and development of PCa is crucial for its diagnosis and treatment. Although the molecular mechanisms underlying PCa progression remain unclear, genetic alterations and signaling pathways have been found to play key roles (Wang et al., 2022b).

The Wnt signaling pathway is a conserved pathway that plays crucial roles in embryonic development, tissue homeostasis, and stem cell maintenance (Zhou et al., 2022a). The Wnt signaling pathway can be categorized into three classes: 1) Wnt/β-catenin signal transduction, 2) Wnt/PCP signal transduction, 3) Wnt/Ca^2+^ signal transduction (Asano et al., 2022). Dysregulation of the Wnt signaling pathway is associated with many diseases, including cancer (Yeh et al., 2019). In PCa, aberrant activation of the Wnt signaling pathway leads to dysregulation of cell proliferation, apoptosis, invasion, and angiogenesis (Koushyar et al., 2022). Understanding the mechanisms underlying Wnt signaling dysregulation can provide valuable insights into the pathogenesis of PCa and elucidate potential therapeutic targets.

Non-coding RNAs (ncRNAs) have been recognized as critical regulatory factors in gene expression and signal transduction in both normal physiology and disease pathogenesis, particularly in PCa (Ferri et al., 2022; Szaflik et al., 2022). ncRNAs are known to execute diverse regulatory roles in cellular processes, despite their inability to encode proteins (Wang et al., 2022a). Recent studies have elucidated several types of ncRNAs, such as microRNAs (miRNAs), long non-coding RNAs (lncRNAs), and circular RNAs (circRNAs), which play key roles in the regulation of the Wnt signaling pathway in PCa (Goodall and Wickramasinghe, 2021; Xue et al., 2022). The alterations in the expression patterns of these ncRNAs at distinct stages of PCa progression indicate their potential as diagnostic and prognostic biomarkers (He et al., 2020; Song et al., 2020). For example, the high expression of the lncRNA CCAT2 and SOX2-OT is associated with the diagnosis and prognosis of PCa, indicating their potential utility as biomarkers (He et al., 2020; Song et al., 2020). Moreover, these ncRNAs affect the characteristics of PCa cells such as proliferation, invasion, migration, and apoptosis; additionally, these ncRNAs can influence the therapeutic response of cancer cells by regulating the Wnt signaling pathway (He et al., 2020; Song et al., 2020; Wang et al., 2021). Therefore, Wnt signaling pathway-related ncRNAs are promising prospects as therapeutic targets for PCa.

In conclusion, our review presents a comprehensive elucidation of the intricate interplay between ncRNAs and the Wnt signaling pathway in the context of PCa occurrence, progression, and therapeutic approaches. Significantly, we underscore the profound implications of ncRNAs as promising diagnostic and prognostic biomarkers, accentuating their pivotal role in modulating PCa aggressiveness and therapeutic response. Moreover, we delve deep into the immense potential of targeting ncRNAs as therapeutic interventions for PCa, exploring a plethora of strategic avenues. Finally, we address the contemporary challenges encountered in this ever-evolving field.

## 2 Wnt signaling pathway and its role in PCa development

**FIGURE 1:**
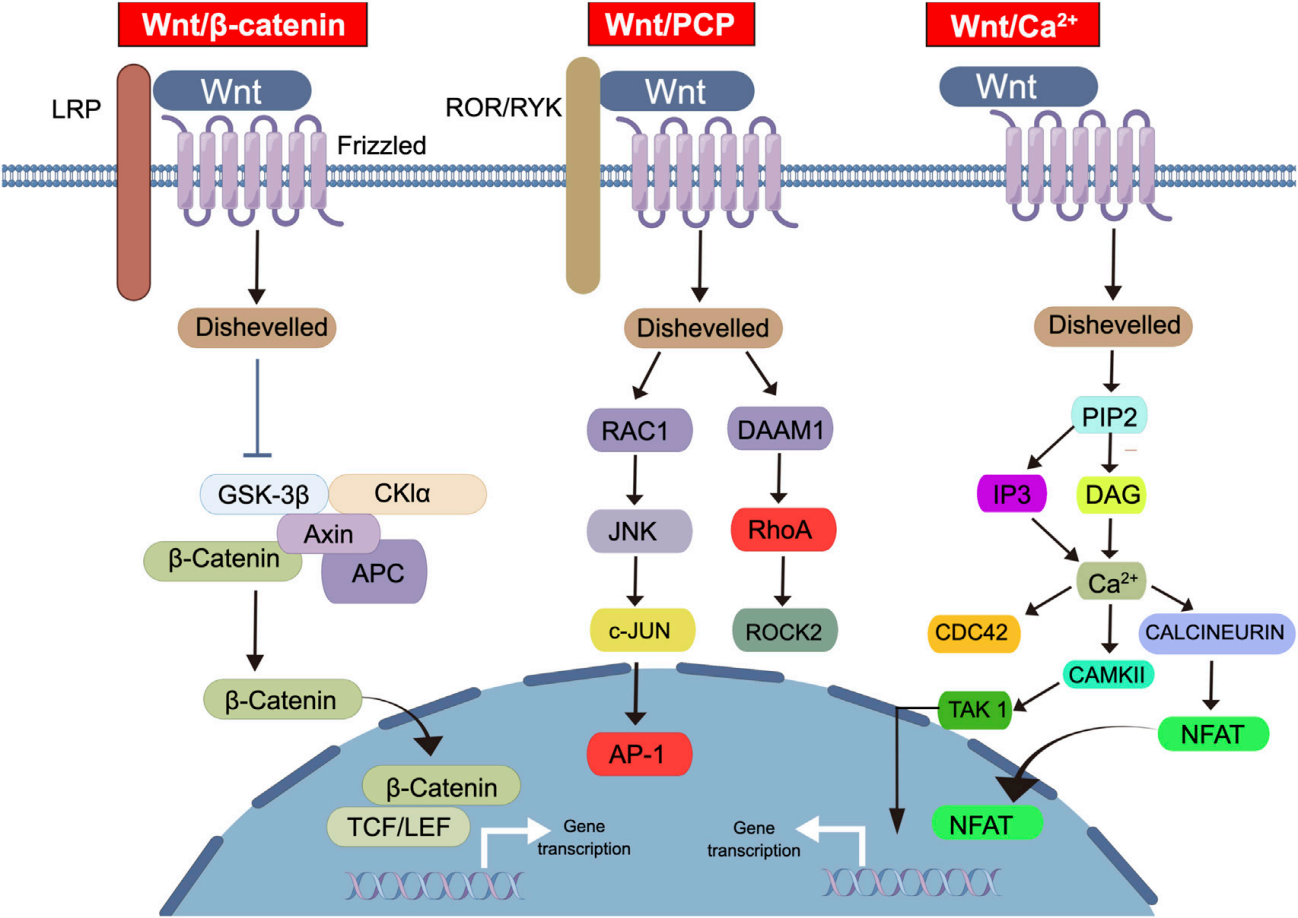
The diverse types and molecular mechanisms of the Wnt signaling pathway. This diagram illustrates the classification and members of the Wnt signaling pathway. The Wnt signaling pathway can be categorized into three classes: (1) Wnt/β-catenin signal transduction, (2) Wnt/PCP signal transduction, (3) Wnt/Ca^2+^ signal transduction. In the Wnt/β-catenin pathway, Wnt ligands bind to Frizzled receptors and LRP co-receptors, leading to the activation of the disheveled protein and subsequent inactivation of Axin protein. This results in the accumulation of β-catenin in the cytoplasm, its translocation into the nucleus, and binding to TCF/LEF transcription factors, ultimately inducing downstream gene expression. Wnt/PCP signaling pathway involved in cell polarity, tissue morphogenesis, cell adhesion, and directional migration through JNK and Rho GTPase signaling pathways. The Wnt/Ca^2+^ signaling pathway modulates gene expression and cell behavior by regulating intracellular Ca^2+^ levels. Abbreviations: Wnt, Wingless-related integration site; LRP, Low-density lipoprotein receptor-related protein; GSK3β, Glycogen Synthase Kinase 3 beta; CKIα, Casein kinase I alpha; Axin, Axis inhibitor; APC, Adenomatous Polyposis Coli; TCF, T-cell factor; LEF, Lymphoid enhancer-binding factor; RAC1, Ras-related C3 botulinum toxin substrate 1; DAMM1, Disheveled-associated activator of morphogenesis 1; JNK, c-Jun N-terminal kinase; RhoA, Ras homolog family member A; c-JUN, Cellular Jun oncogene; ROCK2, Rho-associated coiled-coil kinase 2; AP-1, Activator protein 1; PIP2, Phosphatidylinositol 4,5-bisphosphate; IP3, Inositol trisphosphate; DAG, Diacylglycerol; CDC42, Cell division control protein 42 homolog; TAK1, Transforming growth factor-beta-activated kinase 1; CAMKII, Ca2^+^/calmodulim-dependent protein kinase II; NFAT, Nuclear factor of activated T-cells.

The Wnt signaling pathway is a complex intracellular signaling network that is involved in the regulation of cell fate, proliferation, and differentiation in many tissues (Figure 1) (Asano et al., 2022; Zhou et al., 2022a). Dysregulation of this pathway is associated with the occurrence and progression of many cancers, including PCa (Nusse and Clevers, 2017; Pisano et al., 2021). The Wnt signaling pathway can be categorized into the following two types: canonical and non-canonical (Rim et al., 2022). The canonical Wnt/β-catenin pathway operates through a mechanism whereby the binding of the Wnt protein to its receptor Frizzled triggers the activation of the Disheveled protein, leading to the inactivation of Axin protein and ultimately reducing the degradation of β-catenin (Rim et al., 2022). Consequently, β-catenin gradually accumulates in the cytoplasm and subsequently translocates into the nucleus, where it binds to TCF/LEF transcription factors to induce downstream gene expression (Rim et al., 2022). The non-canonical pathway includes the Wnt/PCP and Wnt/Ca^2+^ signaling pathways (Menck et al., 2021; Sarabia-Sanchez et al., 2023; VanderVorst et al., 2023). The Wnt/PCP signaling pathway is primarily involved in the regulation of cell polarity and tissue morphogenesis (VanderVorst et al., 2023). Additionally, it regulates cell adhesion and directional migration by activating the JNK and Rho GTPase signaling pathways (VanderVorst et al., 2023). The Wnt/Ca^2+^ signaling pathway primarily modulates gene expression and cell behavior by regulating intracellular Ca^2+^ levels (Sarabia-Sanchez et al., 2023). Moreover, it plays a critical role in embryonic nervous system development, cell polarity, and glial cell differentiation (Sarabia-Sanchez et al., 2023). The Wnt/Ror signaling pathway was recently discovered, and its function remains to be comprehensively elucidated (Menck et al., 2021). However, it has been shown to induce the formation and repair of synapses in neurons through certain Wnt ligands (Menck et al., 2021).

The Wnt signaling pathway is activated through the interaction of ligands and receptors on cell surfaces, leading to subsequent signal transduction through intracellular signaling molecules such as β-catenin and TCF/LEF transcription factors (Zhou et al., 2022b). In PCa, aberrant activation of the Wnt signaling pathway leads to dysregulation of cell proliferation, apoptosis, invasion, and angiogenesis (Khurana and Sikka, 2019; Lin et al., 2020). This dysregulation often results from mutations or alterations in key components of the Wnt signaling pathway, such as adenomatous polyposis coli (APC), Axin, and β-catenin, which promote cell cycle progression by activating cyclin D1 and c-myc and enhancing the induction effect exerted by TGF-β signaling (Reya and Clevers, 2005; Zhong et al., 2020; Yu et al., 2021). In addition, these components possibly promote neovascularization and tumor invasion by increasing the expression of VEGF, MMP-9, and IL-8 (Vallee and Lecarpentier, 2018).

Aberrant activation of the Wnt signaling pathway can serve as a biomarker for PCa (Yeh et al., 2019; Pisano et al., 2021). The expression of β-catenin protein in PCa tissue is significantly increased, and mutations and dysregulation of Wnt signaling pathway-related genes can also lead to the aberrant activation of this pathway (Yeh et al., 2019; Pisano et al., 2021). Distinct mutations in the Wnt signaling pathway genes are associated with specific subtypes of PCa. For example, APC mutations are frequently observed in early-stage and low-grade PCa, whereas β-catenin mutations are observed in advanced-stage and high-grade PCa (Desai et al., 2022; Mangolini et al., 2022). Furthermore, in metastatic PCa, an increased proportion of activation mutations in the Wnt/β-catenin signaling pathway-related genes is observed (Desai et al., 2022; Mangolini et al., 2022). The aberrant activation of the Wnt signaling pathway is also associated with the staging and metastasis of PCa (Yeh et al., 2019; Pisano et al., 2021). The classical Wnt/β-catenin signaling pathway is activated in late-stage PCa and bone metastatic PCa, facilitating cell proliferation and drug resistance (Yeh et al., 2019; Pisano et al., 2021).

Multiple components of the Wnt signaling pathway are potential targets in PCa therapeutic interventions (Koushyar et al., 2022). The inhibition of the Wnt signaling pathway in PCa may be crucial for its prevention and treatment. Few studies have investigated methods for inhibiting the Wnt/β-catenin signaling pathway (Brown, 2005; Park and Kim, 2023). For example, researchers have developed and evaluated the efficacy of Protac/molecular glue, antibody-drug conjugates, and anti-sense oligonucleotides in inhibiting the Wnt signaling pathway in preclinical models and clinical trials (Park and Kim, 2023). Additionally, RNA interference technology has shown promising results in preclinical models (Brown, 2005). However, the investigation of these methods is at its nascent stages, and further investigation is necessary to ascertain their efficacy in clinical treatment. Epigenetic alterations also regulate the activity of the Wnt signaling pathway in PCa (Xiong et al., 2009; Eismann et al., 2023). Alterations in DNA methylation and histone modification patterns frequently occur in PCa cells, leading to alterations in gene expression and the activity of the Wnt signaling pathway (Xiong et al., 2009; Eismann et al., 2023). Therefore, directing therapeutic interventions toward these epigenetic modifications may be a viable approach.

The crosstalk between the Wnt signaling pathway and other signaling pathways, such as the androgen receptor (AR) signaling pathway, is gaining recognition as a key factor in the pathogenesis of PCa (Pisano et al., 2021). This presents a challenge in the targeting of the Wnt signaling pathway in PCa treatment (Pisano et al., 2021). The critical role of AR signaling in PCa progression is well-known, and recent studies indicate that Wnt and AR signals interact in complex ways, regulating each other’s activities (Pisano et al., 2021). Thus, disrupting this crosstalk may be necessary to achieve desired treatment outcomes. Overall, a comprehensive examination of the Wnt/β-catenin signaling pathway may provide novel insights and avenues for the diagnosis, treatment, and prevention of PCa.

## 3 The involvement of NcRNAs in modulating the Wnt signaling pathway in PCa

**FIGURE 2:**
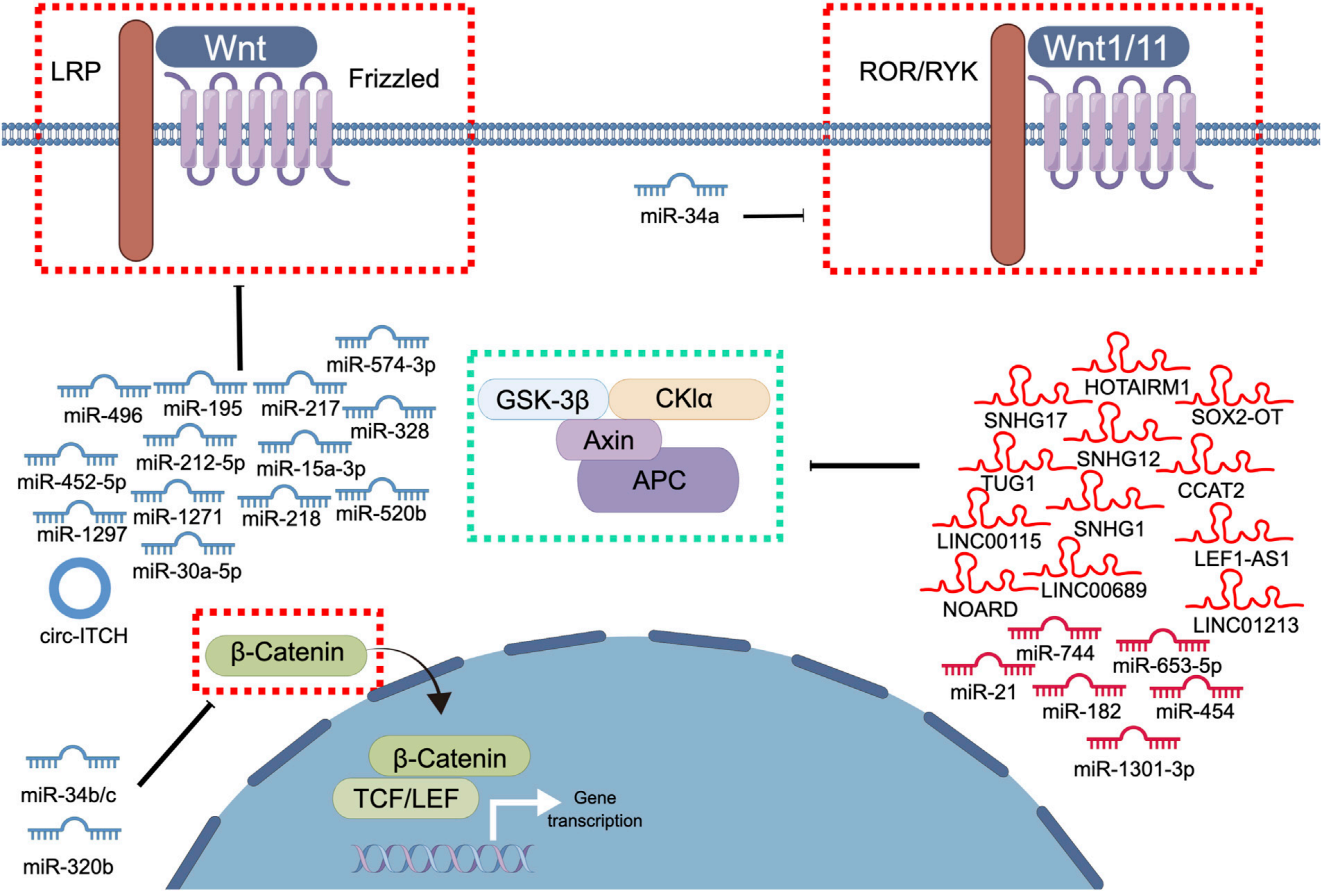
The involvement of ncRNAs in modulating the Wnt signaling pathway in prostate cancer. This diagram illustrates the involvement of ncRNAs in modulating the Wnt signaling pathway in PCa. Blue represents the role of suppressing the Wnt signaling pathway. Red represents roles that facilitate the Wnt signaling pathway.

The dysregulation of ncRNAs has been associated with the occurrence and progression of PCa (Ramnarine et al., 2019). Recent studies have elucidated several types of ncRNAs, such as miRNAs, lncRNAs, and circRNAs, which play crucial roles in PCa by regulating the Wnt signaling pathway (Figure 2). Various types of ncRNAs interact with each other and with other regulatory factors, such as transcription factors, and induce epigenetic modifications, to activate the Wnt signaling pathway (Ramnarine et al., 2019; Orafidiya et al., 2022). For example, the lncRNA SOX2-OT downregulates transcription factor 7-like (TCF7L), a negative regulator of the Wnt signaling pathway, thereby activating the pathway (Song et al., 2020). Similarly, miR-182 regulates the Wnt signaling pathway by targeting APC and glycogen synthase kinase 3 beta (GSK3β), which are two key components of the destruction complex that regulates β-catenin stability (Wang et al., 2018). ncRNAs also activate the Wnt signaling pathway by directly regulating the expression of Wnt signaling pathway-related genes. For example, the lncRNA TUG1 is highly expressed in PCa tissues and cells and promotes cell proliferation, migration, and invasion through the miR-496/Wnt/β-catenin axis (Xiu et al., 2020). miRNAs, such as miR-653-5p, miR-182, miR-1301-3p, and miR-454, also activate the Wnt/β-catenin signaling pathway and promote the malignant progression of PCa cells (Guan et al., 2017; Fu et al., 2018; Wang et al., 2018; Fu et al., 2019). Although many ncRNAs activate the Wnt signaling pathway to promote PCa progression, certain ncRNAs inhibit the Wnt signaling pathway to exert an anti-cancer effect on PCa (Dong et al., 2020). For example, the well-characterized tumor suppressor miR-34a targets multiple Wnt signaling pathway-related genes, including those of Wnt1, LEF1, and β-catenin (Dong et al., 2020).

Despite the aforementioned findings, the molecular mechanism of the regulation of the Wnt signaling pathway by ncRNAs in PCa is not entirely understood, and additional investigations are required to elucidate the complex interplay between various types of ncRNAs and other regulatory factors that activate or inhibit the Wnt signaling pathway in PCa. Furthermore, the investigation of ncRNAs poses technical challenges, owing to their low abundance and high sequence variability (Huang et al., 2023). Advances in next-generation sequencing technologies and bioinformatics tools have improved our ability to identify and validate ncRNA targets and their interactions with the Wnt signaling pathway (Mattick et al., 2023).

## 4 Type and role of Wnt signaling pathway-related NcRNAs in PCa progression

The Wnt signaling pathway plays a key role in the progression of PCa, with ncRNAs as crucial regulatory factors of this pathway (Pakula et al., 2017; Sonawala et al., 2022). Recent studies have elucidated several types of Wnt signaling pathway-related ncRNAs, including miRNAs, lncRNAs, and circRNAs, which exhibit abnormal expression in PCa and are closely linked to its progression (Table 1).

**TABLE 1:** Type and role of Wnt signaling pathway-related ncRNAs in PCa progression.

	ncRNA	Role	Function	Signaling network	Ref.
miRNA	miR-34a	Suppressor	Inhibit proliferation, invasion, migration	Wnt1	Dong et al. (2020)
			Promote apoptosis		
			Inhibit EMT	LEF1	Liang et al. (2015)
			Inhibit proliferation, invasion, migration, and EMT	TCF7, BIRC5	Chen et al. (2015)
	miR-34b/c	Suppressor	Inhibit proliferation, invasion, migration, and EMT	β-catenin	Liu et al. (2015)
	miR-1297	Suppressor	Inhibit proliferation and invasion	AEG-1	Liang et al. (2016)
	miR-1271	Suppressor	Inhibit proliferation and invasion	DIXDC1	Zhong et al. (2017)
	miR-218	Suppressor	Inhibit proliferation and invasion	LGR4	Li et al. (2016b)
	miR-520b	Suppressor	Inhibit proliferation and invasion	Capn4	Ren et al. (2018)
	miR-138	Suppressor	Inhibit proliferation, invasion, and migration	—	Yu et al. (2018)
	miR-574-3p	Suppressor	Inhibit proliferation, invasion, and migration	RAC1	Chiyomaru et al. (2013)
	miR-15a-3p	Suppressor	Inhibit proliferation, invasion, and EMT	SLC39A7	Cui et al. (2019)
	miR-320	Suppressor	Inhibit stem cell- like characteristics	β-catenin	Hsieh et al. (2013)
	miR-653-5p	Oncogene	Promote proliferation and invasion	SOX30	Fu et al. (2019)
	miR-182	Oncogene	Promote proliferation, invasion, and migration	GSK-3β, APC, CK1, and Axin	Wang et al. (2018)
			Inhibit apoptosis		
	miR-454	Oncogene	Promote proliferation and invasion	NDRG2	Fu et al. (2018)
	miR-744	Oncogene	Promote proliferation, invasion, and migration	SFRP1, GSK3β, TLE3, and NKD1	Guan et al. (2017)
	miR-21	Oncogene	Inhibit apoptosis	—	Zhou et al. (2016)
	miR-1301-3p	Oncogene	Promote cancer stem cell expansion	GSK3β and SFRP1	Song et al. (2018)
LncRNA	CCAT2	Oncogene	promote proliferation, invasion, migration	miR-217/TCF7L/Wnt/β-catenin	He et al. (2020)
	TUG1	Oncogene	promote proliferation, invasion, migration	miR-496/Wnt/β-catenin	Li et al. (2020a)
	SOX2-OT	Oncogene	promote proliferation, invasion, migration	miR-452-5p/HMGB3/Wnt/β-catenin	Song et al. (2020)
	SNHG12	Oncogene	promote proliferation, invasion, migration	miR-195/Wnt/β-catenin	Wang et al. (2019)
	LINC00115	Oncogene	promote proliferation, invasion	miR-212-5p/FZD5/Wnt/β-catenin	Peng et al. (2021)
	LINC00689	Oncogene	promote proliferation, invasion, migration	miR-496/CTNNB1/Wnt/β-catenin	Meng et al. (2020)
			Inhibit apoptosis		
	NORAD	Oncogene	promote proliferation, invasion, and EMT	miR-30a-5p/RAB11/Wnt/β-catenin	Zhang and Li (2020)
	SNHG1	Oncogene	promote proliferation, invasion, migration	EZH2/Wnt/β-catenin	Chen et al. (2020)
	LncRNA625	Suppressor	Inhibit proliferation and cell cycle	miR-432/Wnt/β-catenin	Li et al. (2017)
			Promote apoptosis		
	HOTAIRM1	Oncogene	promote proliferation	—	Wang et al. (2021)
			Inhibit apoptosis		
	LEF1-AS1	Oncogene	promote proliferation, invasion, angiogenesis in AIPC	miR-328/FZD2/CD44/Wnt/β-catenin	Li et al. (2020d)
	LINC01213	Oncogene	Androgen-independent transformation	—	Luo et al. (2020)
CircRNA	circ-ITCH	Suppressor	Inhibit proliferation, invasion, and migration	—	Li et al. (2020c)

### 4.1 miRNAs

miRNAs are RNA molecules that are 22 nucleotides in length and regulate gene expression by binding to complementary target messenger RNAs (mRNAs) (Lagos-Quintana et al., 2001). The tissue-specific expression of miRNAs provides a premise for their clinical application as diagnostic and prognostic markers in cancer (Kim and Croce, 2021). For example, miR-574-3p is significantly downregulated in PCa tissues, and low expression of miR-574-3p is associated with an advanced tumor stage and a high Gleason score (Chiyomaru et al., 2013). The interactions between miRNAs and the Wnt signaling pathway in PCa are of two types: direct targeting of key genes associated with the Wnt signaling pathway and indirect targeting of the pathway via other genes. Based on their function, miRNAs can be categorized as oncogenic miRNAs or tumor suppressor miRNAs. Most miRNAs, including miR-34a, miR-34b/c, miR-1297, miR-1271, miR-218, miR-520b, miR-138, miR-574-3p, miR-15a-3p, and miR-320, exert tumor-suppressive effects by directly or indirectly inhibiting the Wnt signaling pathway (Chiyomaru et al., 2013; Hsieh et al., 2013; Liu et al., 2015; Li et al., 2016b; Liang et al., 2016; Zhong et al., 2017; Ren et al., 2018; Yu et al., 2018; Cui et al., 2019; Dong et al., 2020). However, a few miRNAs, such as miR-653-5p, miR-182, miR-1301-3p, and miR-454, promote malignant progression of PCa cells, such as proliferation, invasion, and migration, by activating the Wnt signaling pathway and targeting tumor suppressor genes (Guan et al., 2017; Fu et al., 2018; Wang et al., 2018; Fu et al., 2019). Simultaneously, miRNAs can also suppress stemness in PCa by inhibiting the Wnt signaling pathway (Hsieh et al., 2013; Zhou et al., 2016; Song et al., 2018). For example, miRNAs, such as miR-320 and miR-1301-3p, inhibit the activation of the Wnt signaling pathway and suppress the proliferation of PCa stem cells (Hsieh et al., 2013; Song et al., 2018). In addition, certain naturally active chemical substances have been found to exert therapeutic effects on PCa by regulating miRNAs and the Wnt signaling pathway. For example, urolithin, an active metabolite produced by human colonic microbiota, has been found to inhibit miR-21 and its downstream Wnt signaling pathway, thereby promoting apoptosis of PCa cells and inhibiting tumor growth (Zhou et al., 2016). Therefore, miRNAs and the Wnt signaling pathway may become notable therapeutic targets in PCa treatment, and their in-depth examination may reveal the pathogenesis of PCa and aid in the development of novel drugs against PCa.

### 4.2 LncRNAs

LncRNAs are RNA molecules that are 200 nucleotides in length and do not encode proteins (Szaflik et al., 2022). Although most of the genome is comprised of ncRNAs that are encoded by “junk DNA,” they were originally considered to lack physiological functions (Huang et al., 2023). However, as the potential roles of lncRNAs in biological processes have been unveiled, the aforementioned notion has gradually changed (Wang et al., 2022a). LncRNAs regulate gene expression at various levels, i.e., chromatin, transcriptional, and post-transcriptional levels (Bhattacharjee et al., 2023; Petrone et al., 2023). Mounting evidence suggests that lncRNAs play crucial roles in PCa cell invasion, migration, and apoptosis and in castration-resistant PCa (CRPC), thereby affecting the proliferation, migration, and response of cancer cells to treatment (Li et al., 2020d; Song et al., 2020; Liu et al., 2021; Tan et al., 2021; Wu et al., 2021). LncRNAs can serve as signals, baits, or scaffolds to modulate cellular functions (Yang et al., 2020; Yan and Bu, 2021). Similar to miRNAs, lncRNAs may exert tumor-suppressive or oncogenic effects, contingent upon their category and mode of regulation. For example, lncRNAs such as TUG1, SOX2-OT, LINC04080, LINC00115, LINC00689, NORAD, SNHG1, and LEF1-AS1 activate the Wnt signaling pathway and promote tumor growth and metastasis by regulating other ncRNAs or proteins (Wang et al., 2019; Li et al., 2020a; Chen et al., 2020; Meng et al., 2020; Song et al., 2020; Zhang and Li, 2020; Peng et al., 2021). Conversely, lncRNAs such as lncRNA625 and HOTAIRM1 inhibit the Wnt signaling pathway to suppress cancer, promote cell apoptosis, and inhibit cell proliferation in PCa (Li et al., 2017; Wang et al., 2021). Investigation of the role of lncRNA625 in cancers has revealed that it promotes tumor development in esophageal carcinoma; however, it exerts a significant tumor-suppressive effect on PCa, suggesting that lncRNA625 could potentially serve as a therapeutic target for PCa (Li et al., 2017; Guo-Wei et al., 2019). Given the lack of effective PCa treatments, the investigation of treatments based on Wnt signaling pathway-related lncRNAs is of great significance for developing novel therapeutic strategies for PCa. Therefore, in-depth research on lncRNAs is expected to yield novel treatment options for patients with PCa. In addition, Wnt signaling pathway-related lncRNAs may have notable implications for prognosis evaluation and diagnostic positioning for PCa, with diverse potential applications.

### 4.3 CircRNAs

CircRNAs were first discovered in plant viruses and the Sendai virus through electron microscopy in 1976 (Kolakofsky, 1976). However, it is a widely held belief that circRNAs are the result of splicing errors and exhibit low expression levels (Memczak et al., 2013). With the advancement of bioinformatics and sequencing technologies, various types of circRNAs have been implicated in tumors (Zhou et al., 2021). For example, cir-znf215 has been found to promote the growth and metastasis of cholangiocarcinoma by inhibiting the AKT pathway (Liao et al., 2023). circRNAs also exhibit tissue- and cell-specific expression (Wang et al., 2023). Therefore, circRNAs could potentially serve as diagnostic and prognostic markers as well as therapeutic targets for PCa. cir-ITCH is typically downregulated in PCa tissues and cell lines compared with normal adjacent tissues and normal RWPE-1 cells, indicating the potential of cir-ITCH as a diagnostic and prognostic marker for PCa (Li et al., 2020c). Moreover, cir-ITCH exerts an anti-cancer effect on PCa by inhibiting the Wnt signaling pathway (Li et al., 2020c). In addition, circRNAs interact with ncRNAs and regulate each other’s expression levels (Li et al., 2020c; Ghafouri-Fard et al., 2021). For example, mutual inhibition of expression has been observed between cir-ITCH and miR-17 in PCa (Li et al., 2020c; Ghafouri-Fard et al., 2021).

## 5 Molecular mechanisms and functions of Wnt signaling pathway-related NcRNAs in PCa

### 5.1 Invasion and migration

**FIGURE 3:**
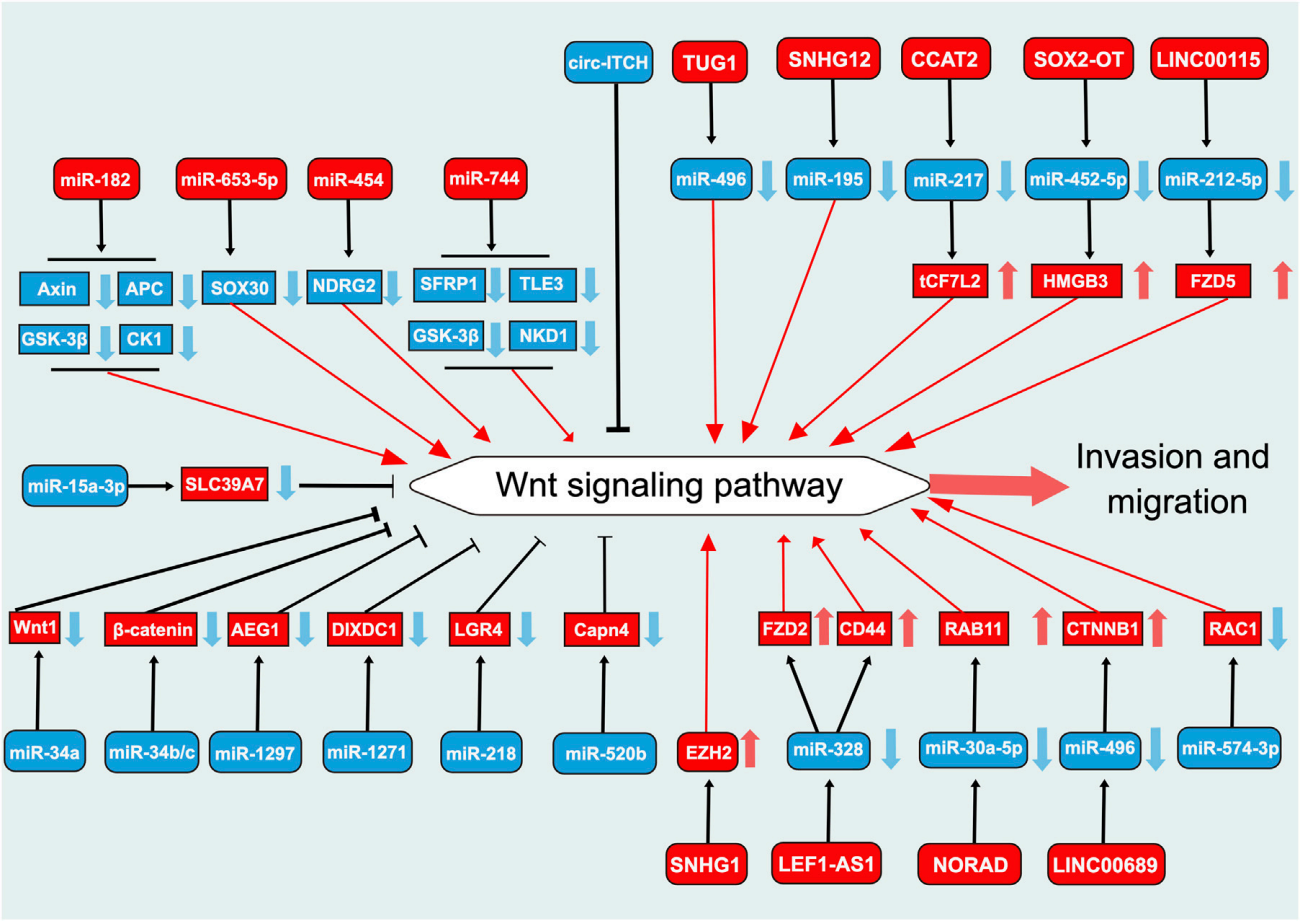
Role of Wnt signaling pathway-related ncRNAs in the regulation of invasion and migration in prostate cancer. This diagram illustrates the role of Wnt signaling pathway-related ncRNAs in the regulation of invasion and migration in PCa. Blue represents the role of suppressing the Wnt signaling pathway. Red represents roles that facilitate the Wnt signaling pathway.

Metastasis is the primary cause of most cancer-related deaths (Fares et al., 2020). Invasion and migration are critical steps in the cascade of tumor metastasis (Fares et al., 2020). miR-34a is a key regulatory factor in tumor suppression, modulating the expression of numerous target proteins involved in cell cycle, differentiation, epithelial-to-mesenchymal transition (EMT), and apoptosis, among others, and antagonizing processes such as cancer cell activity, stemness, metastasis, and chemoresistance (Misso et al., 2014). The expression of miR-34a is significantly lower in PCa tissues than in normal tissues (Lichner et al., 2015). The overexpression of miR-34a significantly reduces the proliferation and migration abilities of PCa cell lines, sush as PC3 cells (Lichner et al., 2015) (Figure 3). These effects are achieved by inhibiting the Wnt signaling pathway through the regulation of Wnt1 transcriptional activity (Dong et al., 2020). Notably, bones are the most common site of metastasis in PCa (Coleman et al., 2020). In PCa exhibiting activated Ras signaling, bone metastasis associated with low expression of miR-34a has been observed (Chen et al., 2015). miR-34a knockdown has been observed to induce the expression of TCF7 and BIRC5 by activating the Wnt signaling pathway, thereby promoting cell survival (Chen et al., 2015). These findings suggest that miR-34a could serve as a potential target in the treatment of metastatic PCa.

Li et al. demonstrated that miR-1297 directly targets the 3′-untranslated region of AEG-1 and regulates its mRNA and protein expression levels (Liang et al., 2016). In addition, they found that miR-1297 inhibits the Wnt signaling pathway by targeting AEG-1 in PCa, thereby suppressing cell proliferation and invasion (Liang et al., 2016). DIXDC1 is involved in the regulation of the proliferation and invasion of various tumors (Zhong et al., 2017; Xin et al., 2018). In PCa, it can be directly targeted and inhibited by miR-1271 (Zhong et al., 2017). MiR-1271 exhibits low expression in PCa and inhibits cell proliferation, invasion, and Wnt signal transduction by targeting DIXDC1 (Zhong et al., 2017).

Epidemiological and histopathological evidence suggests a correlation between inflammation and PCa incidence (De Nunzio et al., 2011). LGR4, which is induced by IL-6 during cancer progression, has been recently identified as a response gene associated with PCa progression (Liu et al., 2013). Yang et al. found that miR-218 directly targets LGR4 to inhibit the Wnt signaling pathway in LNCaP-IL-6^+^ cells during IL-6-induced PCa cell progression, thereby suppressing cell proliferation, cell cycle progression, and invasion (Li et al., 2016b). CapnS1 has been found to have a negative correlation with disease progression in various solid tumors (Zheng et al., 2020). In PCa, CapnS1 expression is regulated by miR-520b, and it exerts an oncogenic effect by promoting Wnt signal transduction (Ren et al., 2018). miR-520b is significantly downregulated in PCa (Ren et al., 2018). Inhibition of CapnS1 by miR-520b suppresses the growth and invasion of PCa cells associated with the downregulation of Wnt signal transduction (Ren et al., 2018). miR-138 has been observed to be downregulated in invasive PCa cell lines and promote PCa cell invasion and migration through the Wnt signaling pathway, whereas its overexpression has been observed to suppress these functions (Yu et al., 2018). miR-574-3p is significantly downregulated in PCa, and its low expression is associated with advanced tumor stage and a high Gleason score (Chiyomaru et al., 2013). The overexpression of miR-574-3p significantly inhibits the proliferation, migration, and invasion of PCa cells, which is associated with the inhibition of the Wnt signaling pathway via RAC1 (Chiyomaru et al., 2013). SOX30 is a recently identified cancer-related member of the SOX family that has a significant role in various types of cancer (Fu et al., 2019). miR-653-5p is highly expressed in PCa tissues and promotes the proliferation and invasion of PCa cells by targeting and upregulating β-catenin expression via SOX30 and activating the Wnt signaling pathway (Fu et al., 2019). miR-182 expression is higher in PCa tissues than in non-cancerous tissues, and miR-182 significantly activates the Wnt signaling pathway by targeting multiple negative regulators of Wnt signaling, thereby promoting cell proliferation, colony formation, migration, and invasion (Wang et al., 2018). miR-454 is highly expressed in PCa tissues and cell lines and promotes PCa cell proliferation and invasion by upregulating the Wnt signaling pathway, which is achieved by inhibiting NDRG2 expression (Wei et al., 2020). miR-744 significantly activates the Wnt signaling pathway by targeting multiple negative regulators of the pathway and promotes PCa cell proliferation, migration, and invasion (Guan et al., 2017).

TUG1, a 7.1 kb lncRNA, was first discovered to be upregulated in mouse retinal cells in response to taurine treatment (Young et al., 2005). TUG1 is highly expressed in PCa tissues and cells and promotes cell proliferation, migration, and invasion through the miR-496/Wnt/β-catenin axis (Li et al., 2020b; Xiu et al., 2020). LncRNA SOX2-OT plays crucial roles in psychiatric disorders, cancer, and diabetic complications (Li et al., 2020c). In PCa tissues and cells as well, SOX2-OT is highly expressed. Regulation of the miR-452-5p/HMGB3 axis and inactivation of the Wnt signaling pathway have been shown to inhibit PCa cell proliferation and metastasis, thereby suppressing tumor growth *in vivo* (Song et al., 2020). SNHG12, also known as LINC04080, is a lncRNA spanning approximately 1867 nucleotides and is located in the 1p35.3 region (Lan et al., 2017). In PCa, SNHG12 expression is upregulated in serum and tissues and is associated with RFS, biochemical recurrence, and Gleason scores of 8–10 in patients (Wang et al., 2019). This lncRNA activates the Wnt signaling pathway through the sponging effect of miR-195, thereby promoting cell proliferation, invasion, and migration in PCa (Song et al., 2019). LINC00115 was first identified as a notable pro-cancer lncRNA in lung cancer (Li et al., 2016a). In PCa, it is highly expressed in tissues and closely associated with a poor prognosis (Peng et al., 2021). LINC00115 promotes PCa cell proliferation and invasion by targeting the miR-212-5p/FZD5/Wnt axis (Peng et al., 2021). LINC00689, first found to be associated with obesity susceptibility genes in the Han Chinese population of northern China, exerts a pro-cancer effect in multiple solid tumors, including gastric cancer, breast cancer, and liver cancer (Liu et al., 2019b; Du et al., 2020; Lu et al., 2020). This lncRNA activates the Wnt signaling pathway by regulating miR-496/CTNNB1, thereby promoting PCa cell proliferation, migration, and invasion (Meng et al., 2020). LEF1 is a key component of the Wnt/β-catenin signaling pathway. It is highly expressed in PCa and is associated with its malignant progression (Fakhr et al., 2021). The recently identified lncRNA LEF1-AS1 is encoded by the LEF1 locus and is associated with poor prognosis in multiple cancer types (Li et al., 2020d). LEF1-AS1 promotes PCa metastasis and serves as a competing endogenous RNA (ceRNA) for miR-328, thereby modulating Wnt/β-catenin pathway activity by regulating FZD2 and CD44, ultimately promoting androgen-independent PCa (AIPC) cell proliferation, migration, invasion, angiogenic ability, and tumor growth (Li et al., 2020d). In addition, lncRNAs also play crucial roles in PCa metastasis by directly binding to EZH2 (Chen et al., 2020). For example, both the lncRNAs SNHG1 and EZH2 are highly expressed in PCa tissues and cells, and their expression is positively correlated. SNHG1 regulates the Wnt signaling pathway through the EZH2 gene, modulating PCa cell proliferation, invasion, and migration (Chen et al., 2020). These findings further enrich our understanding of the mechanism of action of Wnt signaling pathway-related lncRNAs in PCa. The aforementioned lncRNAs are associated with the Wnt signaling pathway and play vital roles in the growth and progression of PCa, thereby presenting as novel targets for PCa treatment.

In addition to the aforementioned Wnt signaling pathway-related lncRNAs, the role of the lncRNA CCAT2 in PCa metastasis should be considered (Zheng et al., 2016; He et al., 2020). Studies have shown that complex feedback loops exist between CCAT2 and the Wnt signaling pathway (Zheng et al., 2016; He et al., 2020). Moreover, CCAT2 plays a key role in the invasion and migration of PCa (Zheng et al., 2016; He et al., 2020). Notably, CCAT2 is not only aberrantly expressed in PCa but also exhibits similar expression patterns in many other cancers (Ling et al., 2013). In colorectal cancer, it is a downstream target of the Wnt signaling pathway, indicating that TCF7L2 is also involved in this feedback loop (Ling et al., 2013). Therefore, an in-depth investigation of the role of lncRNAs in the Wnt signaling pathway is of great significance for the prognosis and treatment of cancer as well as the recovery from cancer. In summary, the key role of the lncRNA CCAT2 in PCa metastasis indicates its potential as a therapeutic target.

In contrast to miRNAs and lncRNAs, circRNAs associated with the Wnt signaling pathway in PCa have been the subject of comparatively less investigation. The expression of cir-ITCH is downregulated in PCa tissues and cell lines (Li et al., 2020c), and its overexpression significantly inhibits the proliferation, migration, and invasion of human PCa cells. Cir-ITCH and miR-17 function as mutual expression inhibitory factors (Li et al., 2020c). Cir-ITCH contributes to the suppression of PCa progression by inhibiting the Wnt/β-catenin signaling pathway, which may be achieved through the inhibition of miR-17 (Li et al., 2020c).

### 5.2 EMT

**FIGURE 4:**
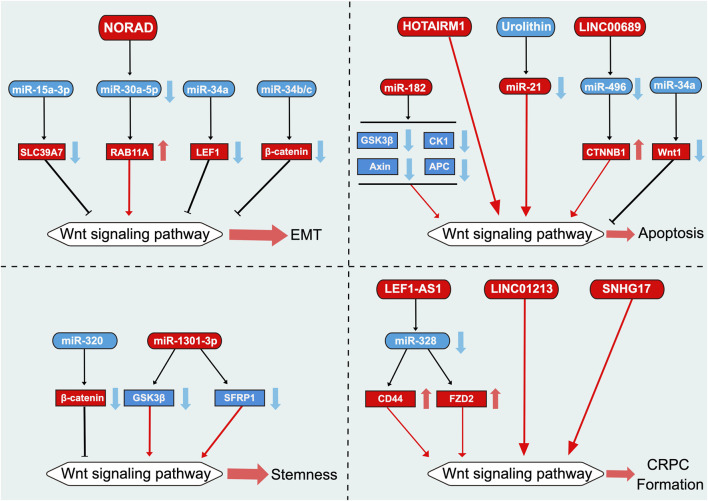
Role of Wnt signaling pathway-related ncRNAs in the regulation of EMT, apoptosis, stemness, and CRPC formation in prostate cancer. This diagram illustrates the role of Wnt signaling pathway-related ncRNAs in the regulation of EMT, apoptosis, stemness, and CRPC formation in PCa. Blue represents the role of suppressing the Wnt signaling pathway. Red represents roles that facilitate the Wnt signaling pathway.

The metastasis of solid tumors is also influenced by the characteristics and plasticity of cancer cells, such as EMT (Babaei et al., 2021). During EMT, epithelial cells transform into highly mobile mesenchymal cells, thereby increasing the migration ability of cancer cells (Hao et al., 2019). Inhibiting EMT is key to preventing cancer metastasis and improving prognosis (Fedele et al., 2022). miR-15a-3p is downregulated in PCa tissues and cell lines, whereas its overexpression inhibits cell proliferation, invasion, and EMT by downregulating the Wnt signaling pathway, with SLC39A7 as its direct downstream target (Cui et al., 2019) (Figure 4). LEF1 is a key transcription factor in the Wnt signaling pathway that regulates cell proliferation and invasion (Fakhr et al., 2021). miR-34a can regulate the level of LEF1 to inhibit EMT in PCa cells (Liang et al., 2015). Additionally, two other members of the miR-34 family, namely miR-34b/c, when overexpressed, can target β-catenin mRNA expression, thereby inhibiting cell migration and EMT in PCa (Liu et al., 2015).

The lncRNA NORAD exerts a pro-cancer effect in melanoma, pancreatic cancer, and glioblastoma (Soghli et al., 2021). In PCa as well, NORAD is highly expressed in cells and tissues and promotes cell proliferation, invasion, and EMT (Zhang and Li, 2020; Hu et al., 2021; Fletcher et al., 2022). miR-30a-5p attenuates NORAD-mediated promotion of cell proliferation, invasion, and EMT by targeting RAB11A (Zhang and Li, 2020).

### 5.3 Apoptosis

Cell apoptosis is a key self-regulation mechanism in multicellular organisms, serving to eliminate unwanted or abnormal cells (Kerr et al., 1972). Dysregulation of cell apoptosis has been implicated in various diseases, including cancer, autoimmune diseases, cardiovascular diseases, and neurological diseases (Chen et al., 2021). In recent years, a growing body of evidence has shown that ncRNAs play a crucial role in PCa cell apoptosis (Tamtaji et al., 2021). The Wnt signaling pathway is associated with PCa cell apoptosis, and ncRNAs associated with this pathway also play key roles in PCa cell apoptosis (Tamtaji et al., 2021). The overexpression of miR-34a inhibits the Wnt signaling pathway by regulating the transcriptional activity of Wnt1, thereby significantly increasing the rate of cell apoptosis (Dong et al., 2020) (Figure 4). Compared with non-cancerous tissues, PCa tissues exhibit upregulated expression of miR-182 (Wang et al., 2018). The upregulation of miR-182 activates the Wnt signaling pathway by targeting negative regulatory factors of the pathway, such as GSK3β, APC, CK1, and Axin, ultimately inhibiting cell apoptosis (Wang et al., 2018). Urolithin, a bioactive metabolite derived from ellagic acid, has been observed to inhibit miR-21 and its downstream Wnt/β-catenin signaling pathway to reduce cell viability and promote caspase-dependent cell apoptosis in DU145 cells (Zhou et al., 2016; Singh et al., 2019).

LINC0689 exerts a pro-oncogenic effect in multiple parenchymal tumors, where its expression is elevated (Liu et al., 2019b; Du et al., 2020). LINC00689 is upregulated in end-stage PCa tissues and inhibits apoptosis through miR-496/CTNNB1 (Meng et al., 2020). In addition, the lncRNA HOTAIRM1 is highly expressed in mature bone marrow cells (Zhang et al., 2014). Silencing HOTAIRM1 in PC3 cells promotes PCa cell apoptosis by downregulating the Wnt/β-catenin signaling pathway; however, the exact mechanism remains unknown (Wang et al., 2021).

### 5.4 Stemness

Cancer stem cells play a crucial role in the survival, proliferation, metastasis, and recurrence of tumors (Murota et al., 2022). miR-320 inhibits the activation of the Wnt/β-catenin signaling pathway by targeting β-catenin mRNA expression, thereby suppressing PCa stem cell characteristics such as tumor sphere formation, chemoresistance, and tumorigenicity (Hsieh et al., 2013) (Figure 4). In contrast, miR-320 knockdown significantly enhances the aforementioned characteristics (Hsieh et al., 2013). miR-1301-3p is significantly upregulated in PCa cells and tissues and targets inhibitors of the Wnt signaling pathway, namely GSK3β and SFRP1, thereby promoting the proliferation of PCa stem cells by activating the Wnt/β-catenin signaling pathway (Song et al., 2018).

### 5.5 CRPC formation

Targeting the Wnt/β-catenin signaling pathway may be an attractive therapeutic strategy for treating CRPC (Shafi et al., 2013). The potential of Wnt signaling pathway-related lncRNAs in the treatment of CRPC is currently gaining increasing attention (Yap et al., 2011) (Figure 4). Androgen deprivation therapy has become the mainstay for the treatment of patients with advanced PCa (Shafi et al., 2013). However, most patients eventually progress to CRPC, leading to poor prognosis (Yap et al., 2011). Bone metastasis is a notable issue in patients with CRPC (Beltran et al., 2016; Lin et al., 2020). Luo et al. elucidated that crosstalk between the AR and Wnt/β-catenin signals promotes the androgen-independent transformation of PCa (Luo et al., 2020). Although androgens can inhibit the Wnt/β-catenin signaling pathway in androgen-dependent PCa cells, this inhibitory effect is not observed in AIPC cells (Luo et al., 2020). Moreover, LEF1-AS1 has been observed to promote PCa metastasis through the Wnt/β-catenin signaling pathway and function as a ceRNA for miR-328, thereby regulating the activity of the Wnt/β-catenin signaling pathway by regulating FZD2 and CD44, ultimately enhancing proliferation, migration, invasion, and angiogenic ability of AIPC cells and tumor growth (Li et al., 2020d). The Wnt/β-catenin signaling pathway inhibits AIPC cell proliferation by promoting the cell cycle process and inhibiting apoptosis (Luo et al., 2020). Therefore, targeting the Wnt/β-catenin signaling pathway may be a viable strategy for the treatment of CRPC.

Exosomes are small vesicles that measure approximately 40–160 nm (typically approximately 100 nm) in diameter and originate from endosomes (Pegtel and Gould, 2019). Many studies have demonstrated the importance of several lncRNAs in exosomes across various cancers (Kalluri and LeBleu, 2020). For example, the exosomal lncRNA HOXD-AS1 has been observed to promote the metastasis of PCa through the miR-361-5p/FOXM1 axis (Jiang et al., 2021). Wnt signaling pathway-related lncRNAs loaded in exosomes could potentially serve as diagnostic and therapeutic tools for the treatment of CRPC (Guo et al., 2022). For example, LINC01213 plays a role in the transition of PCa cells from an androgen-dependent to an androgen-independent state (Guo et al., 2022). Additionally, it induces androgen deprivation tolerance by activating the Wnt signaling pathway through exosome-mediated intercellular communication in PCa (Guo et al., 2022). The lncRNA SNHG17 is one of the four significantly upregulated lncRNAs in metastatic PCa and AIPC cells, wherein it promotes tumor cell proliferation, survival, invasion, and resistance to chemotherapy by upregulating the Wnt/β-catenin signaling pathway (Bai et al., 2020; Zhao et al., 2021).

## 6 Wnt signaling pathway-related NcRNAs in the diagnosis and treatment of PCa

Early detection of PCa is crucial for effective treatment and improved survival rates (Sung et al., 2021). For example, in the event of early detection, the five-year survival rate of patients with localized PCa is nearly 100% (Sandhu et al., 2021). In contrast, the median survival duration for patients with metastatic PCa is approximately 3 years (Sandhu et al., 2021). Therefore, early diagnosis of PCa is essential. Additionally, because PCa is prone to metastasis and chemoresistance, it has become one of the leading causes of cancer-related mortality worldwide (He et al., 2022). Due to the lack of symptoms in the early stages of PCa, despite technological advancements, the discovery of novel tumor biomarkers remains crucial (Wang et al., 2022b). This is necessary to address the challenges associated with the diagnosis and treatment of prostate cancer. NcRNAs exhibit tissue-specific expression and are detectable at all stages of PCa development, making them potential biomarkers and therapeutic targets (Mugoni et al., 2022). Mounting evidence suggests that Wnt signaling pathway-related ncRNAs are closely associated with PCa progression (Doghish et al., 2022), rendering them promising biomarkers for the diagnosis, prognosis, and treatment of PCa (Table 2). Therefore, investigating Wnt signaling pathway-related ncRNAs in the context of early diagnosis, prognosis prediction, cancer treatment, and resolution of treatment resistance is an effective strategy to improve the survival of PCa patients.

**TABLE 2:** Clinical applications of ncRNAs and Wnt/β-catenin pathway in PCa.

NcRNA		Expression	Prognosis	Diagnosis	Clinical significance	Ref.
miRNA	miR-34a	Down	Poor	Profitable	Bone metastasis, gleason score	Chen et al. (2015)
	miR-34b/c	Down	—	Profitable	—	Liu et al. (2015)
	miR-1297	Down	—	Profitable	—	Liang et al. (2016)
	miR-1271	Down	—	Profitable	—	Zhong et al. (2017)
	miR-218	Down	Poor	Profitable	Bone Metastasis, TNM stage, T stage, N stage, M stage and gleason score	Li et al. (2016b)
	miR-520b	Down	Poor	Profitable	—	Ren et al. (2018)
	miR-138	Down	Poor	Profitable	N stage, M stage and gleason score	Yu et al. (2018)
	miR-574-3p	Down	Poor	Profitable	T stage and gleason score	Chiyomaru et al. (2013)
	miR-653-5p	High	—	Profitable	—	Fu et al. (2019)
	miR-182	High	—	Profitable	—	Wang et al. (2018)
	miR-454	High	—	Profitable	—	Fu et al. (2018)
	miR-744	High	Poor	Profitable	CRPC progression	Guan et al. (2017)
	miR-15a-3p	Down	—	Profitable	—	Cui et al. (2019)
	miR-21	High	—	Profitable	DDP Chemoresistance, and pathological stage, N stage, capsular invasion, organ confined disease, gleason score, and biochemical recurrence	Zhou et al. (2016)
	miR-320	Down	Poor	Profitable	DDP chemoresistance, serum PSA levels, TNM stage	Hsieh et al. (2013)
	miR-1301-3p	Down	—	Profitable	—	Song et al. (2018)
	miR-425-5p	Down	Poor	Profitable	DDP chemoresistance, residual tumor, T stage, N stage, and TP53 status	Liu et al. (2019a)
LncRNA	CCAT2	High	Poor	Profitable	Histological grade and M stage	He et al. (2020)
	TUG1	High	Poor	Profitable	TNM stage, gleason score, TNM stage, preoperative PSA level, and N stage	Li et al. (2020a)
	SOX2-OT	High	Poor	Profitable	—	Song et al. (2020)
	SNHG12	High	Poor	Profitable	Biochemical recurrence and gleason score 8–10	Wang et al. (2019)
	LINC00115	High	Poor	Profitable	—	Peng et al. (2021)
	LINC00689	High	Poor	Profitable	TNM stage	Meng et al. (2020)
	NORAD	High	Poor	Profitable	Bone metastasis	Zhang and Li (2020)
	SNHG1	High	Poor	Profitable	TNM stage, Gleason Score, N stage, and long-term metastasis mortality rate	Chen et al. (2020)
	LncRNA625	Down	—	Profitable	—	Li et al. (2017)
	HOTAIRM1	High	—	Profitable	—	Wang et al. (2021)
	LEF1-AS1	High	—	Profitable	—	Li et al. (2020d)
	HOXD-AS1	High	Poor	Profitable	Highly expressed in serum exosomes from metastatic PCa patients, Gleason Score, and N stage	Jiang et al. (2021)
	LINC01213	—	—	—	—	Guo et al. (2022)
	HOTTIP	High	Poor	Profitable	DDP chemoresistance, T stage, presence of extra prostatic extension, seminal vesicle invasion, perineural invasion, and the tumor involvement of resection margin	Jiang et al. (2019)
	SNHG17	High	Poor	Profitable	Docetaxel chemoresistance, Histological grade, T stage, N stage, and M stage	Bai et al. (2020), Zhao et al. (2021)
CircRNA	circ-ITCH	Down	Poor	Profitable	T stage, N stage, Gleason score, and surgical margin status	Li et al. (2020c)

### 6.1 Potential PCa diagnostic biomarkers

Early screening and diagnosis of cancer are crucial for patient survival (Sung et al., 2021). The identification of suitable biomarkers has consistently posed a notable challenge in the field of cancer research (Movahedpour et al., 2022; Xie et al., 2022). Wnt signaling pathway-related ncRNAs aid in the early diagnosis of PCa. In patients with PCa, certain Wnt signaling pathway-related ncRNAs, such as SNHG17 and LINC00115, are upregulated (Peng et al., 2021), whereas other ncRNAs, such as miR-34a, are downregulated (Chiyomaru et al., 2013). Additionally, certain ncRNAs are aberrantly expressed in various stages or special subtypes of PCa (Li et al., 2020d; Meng et al., 2020). For instance, LINC00689 is upregulated in end-stage PCa tissues and LEF1-AS1 is significantly overexpressed in AIPC (Li et al., 2020d; Meng et al., 2020). These findings suggest that Wnt signaling pathway-related ncRNAs could potentially serve as diagnostic biomarkers for PCa. Notably, ncRNAs present in plasma will be relatively non-invasive and more convenient as diagnostic tools. Wnt signaling pathway-related lncRNAs loaded in exosomes could also potentially serve as diagnostic and therapeutic tools for PCa (Guo et al., 2022). For example, exosomal LINC01213 can serve as a diagnostic biomarker for CRPC (Guo et al., 2022).

### 6.2 Potential PCa prognostic biomarkers

Patient prognostic information is essential in the process of making informed treatment decisions (Lin and Farooqi, 2021). Mounting evidence suggests that the Wnt signaling pathway-related ncRNAs may hold significant potential for predicting patient prognosis (Chiyomaru et al., 2013; Dong et al., 2020; Xiu et al., 2020; Peng et al., 2021; Zhao et al., 2021). These ncRNAs are closely associated with overall survival, disease-free survival, recurrence-free survival, five-year survival rates, and progression-free survival of patients with PCa (Peng et al., 2021). For example, high expression of LINC00115 is associated with shorter overall survival and recurrence-free survival in patients with PCa (Peng et al., 2021). Additionally, Wnt signaling pathway-related ncRNAs are also associated with other prognostic factors (Chiyomaru et al., 2013; Dong et al., 2020; Xiu et al., 2020; Zhao et al., 2021). For instance, miR-34a is associated with bone metastasis of Ras-activated PCa cells (Dong et al., 2020). Furthermore, miR-574-3p is associated with advanced tumor stage and higher Gleason scores (Chiyomaru et al., 2013). In contrast, high expression of SNHG17 is associated with grade, stage, and metastasis (Zhao et al., 2021). Additionally, TUG1 is associated with Gleason score, clinical stage, preoperative PSA level, and lymph node metastasis (Xiu et al., 2020). These findings have crucial implications for the prognostic assessment and treatment selection in PCa. Therefore, Wnt signaling pathway-related ncRNAs could potentially serve as vital indicators for the prognostic evaluation and treatment selection in PCa.

### 6.3 Potential therapeutic targets

Cancer treatment has long been regarded as one of the most formidable challenges worldwide, despite the progress made in treatment modalities (Sung et al., 2021). Targeted therapy strategies based on ncRNAs have yielded novel insights into cancer treatment (Paunovska et al., 2022). NcRNAs regulate cell proliferation, invasion, migration, apoptosis, and stemness in PCa and conversion of PCa to CRPC by directly or indirectly interacting with the Wnt signaling pathway (Guan et al., 2017; Wang et al., 2018). Therefore, modulating the expression of Wnt signaling pathway-related ncRNAs could be an effective strategy for treating PCa and improving patient prognosis. For example, silencing miR-182 using inhibitors has been observed to significantly reduce the growth of PCa xenograft tumors, whereas silencing miR-744 using short hairpin RNA (shRNA) has been observed to significantly reduce the growth of PCa xenograft tumors (Guan et al., 2017; Wang et al., 2018). However, identifying targeted drugs that modulate ncRNA expression and stably transmit this effect remains a challenge, and necessites an enhanced understanding of the structure and function of Wnt signaling pathway-related ncRNAs. Most lncRNAs and circRNAs function as “sponges” for miRNAs to activate or deactivate the Wnt signaling pathway (Li et al., 2020d). Therefore, the regulation of target miRNA of Wnt signaling pathway-related lncRNA and circRNA or the interfering with upstream lncRNA and circRNA associated with Wnt signaling pathway-related miRNA could also be a viable treatment strategy. For example, miR-496 intervention has been observed to effectively reverse the growth-promoting effect of TUG1 on PCa xenografts (Li et al., 2020d).

Targeting ncRNAs has been considered an attractive strategy for cancer treatment (Damase et al., 2021; Garbo et al., 2022; Zogg et al., 2022). In addition to using the above approach, there are other methods that can be employed to intervene in the expression of ncRNAs involved in the Wnt pathway, which may provide therapeutic benefits for patients (Damase et al., 2021; Garbo et al., 2022; Zogg et al., 2022). In the field of Wnt pathway-related miRNAs, miRNA mimics (miRNA-like dsRNA) have been found to enhance the expression and function of certain miRNAs; meanwhile, antagomiRs can serve as tools to inhibit oncogenic miRNAs associated with the Wnt pathway, thereby blocking the specific functions of these miRNAs (Neumeier and Meister, 2020; Xu et al., 2023). In recent years, strategies based on lncRNAs for cancer treatment have gained widespread recognition. Currently, the main therapeutic approaches for managing lncRNAs involve modulating their expression levels to decrease oncogenic lncRNAs (through RNA interference methods) or increase tumor-suppressive lncRNAs (Garbo et al., 2022). It is worth noting that targeting strategies for lncRNAs need to take into account their cellular localization. Antisense oligonucleotides (ASOs) are the most effective method for targeting nuclear lncRNAs (Adewunmi et al., 2023). However, small interfering RNAs (siRNAs) are preferred for cytoplasmic lncRNAs (Li et al., 2022). Additionally, other strategies such as aptamers, nucleases, and miRNAs can be developed to disrupt lncRNA activit (Damase et al., 2021; Zogg et al., 2022). Due to their wide biological activity and stability, circRNAs have emerged as a potential and powerful therapeutic strategy that can significantly impact cancer occurrence and progression (Zong et al., 2023). However, limiting off-target effects remains a challenge in this field (Loan Young et al., 2023). Addressing this issue, specific carriers for synthetic circRNAs or siRNAs targeting junction sequences could offer substantial benefits to patients. All in all, targeted therapeutic strategies based on ncRNAs hold promise as a novel approach for PCa treatment.

### 6.4 Potential chemoresistance targets

Chemoresistance is a notable concern in cancer treatment, and enhancing chemosensitivity in PCa through ncRNA intervention has become a strategy that is increasingly being recognized and investigated (Chen et al., 2022). Targeting Wnt signaling pathway-related ncRNAs may help reverse chemoresistance in PCa (Liu et al., 2019a). Cisplatin, a platinum-based chemotherapeutic drug commonly used in the treatment of PCa, works by forming covalent bonds with DNA, leading to the formation of DNA cross-links and inhibiting DNA replication and transcription (Li et al., 2021). In PCa, cisplatin plays a role in inhibiting tumor growth by damaging the DNA of cancer cells and triggering apoptosis (programmed cell death) (Dhar et al., 2011). However, the development of resistance to cisplatin remains a major challenge in PCa treatment. Mechanisms underlying cisplatin resistance in PCa include enhanced DNA repair mechanisms, altered drug uptake and efflux, increased drug inactivation, and alterations in cell death pathways (Kalathil et al., 2023). miR-425-5p is downregulated in PCa and is further downregulated in cisplatin-resistant PCa (Liu et al., 2019a). Therefore, upregulating miR-425-5p by targeting the Wnt signaling pathway could potentially enhance the sensitivity of PCa to cisplatin (Liu et al., 2019a). Additionally, HOTTIP, a known oncogene, is upregulated in patients with PCa and PCa cell lines, promoting PCa cell proliferation and reducing sensitivity to cisplatin by activating the Wnt signaling pathway (Jiang et al., 2019). As a member of a class of chemotherapy drugs known as taxanes, docetaxel acts by disrupting microtubule dynamics, ultimately inhibiting cell division and inducing cell death (Sanchez-Hernandez et al., 2023). Docetaxel exerts its anti-cancer effects by targeting rapidly dividing cancer cells, inhibiting tumor growth, and promoting cancer cell death (Sanchez-Hernandez et al., 2023). In the treatment of PCa, docetaxel is particularly effective in advanced or metastatic CRPC (Gebrael et al., 2023). However, similar to cisplatin, resistance to docetaxel of PCa can develop over time. Mechanisms of docetaxel resistance in PCa involve alterations in microtubule dynamics, activation of cell survival pathways, and increased drug efflux (Gebrael et al., 2023). SNHG17 promotes chemotherapeutic resistance to docetaxel in PCa tumor cells by upregulating the Wnt signaling pathway, thereby leading to increased chemoresistance (Zhao et al., 2021). Therefore, modulation of Wnt signaling pathway-related ncRNAs may be an effective strategy to reverse chemoresistance in PCa. However, identifying targeted drugs that can regulate ncRNA expression and stably transmit this effect remains a challenge (Jaiswal et al., 2023). Therefore, an enhanced understanding of the structure and function of Wnt signaling pathway-related ncRNAs can aid the development of novel treatment strategies to reverse chemoresistance in PCa.

## 7 Conclusion

This review provided a comprehensive overview of the role of the Wnt signaling pathway and related ncRNAs (miRNAs, lncRNAs, and circRNAs) in PCa. MiRNAs affect the expression of target genes associated with the Wnt signaling pathway. Additionally, the Wnt signaling pathway establishes feedback mechanisms and functions as an upstream mediator of miRNAs. In most cases, lncRNAs regulate the expression of proteins in the Wnt signaling pathway by serving as sponges for miRNAs. CircRNAs also regulate the expression of the Wnt signaling pathway; however, similar to lncRNAs, they primarily regulate the expression of the Wnt signaling pathway by targeting miRNAs. We also discussed the novel avenues for the development of siRNAs and shRNAs that target the Wnt signaling pathway and their potential clinical applications. However, the limited efficacy of siRNAs and shRNAs *in vivo* has hindered their potential clinical application, necessitating the exploration of more reliable strategies to target ncRNAs.

Although the regulation of the Wnt signaling pathway through ncRNA intervention is a promising avenue for the treatment of PCa, certain issues need to be addressed. First, the Wnt signaling pathway is highly intricate, consisting of 19 distinct types of Wnt-secreted glycoproteins and over 15 types of Wnt receptors in humans, which activate various downstream pathways. Second, the differences and balance between the classical and non-classical Wnt signals are difficult to capture, making targeting the Wnt signaling pathway even more challenging. Third, the Wnt signaling pathway plays a fundamental role in the dynamic balance of systems, such as the digestive and hematopoietic systems; therefore, blocking the Wnt signaling pathway may lead to systemic toxicity. Hence, modulation of the Wnt signaling pathway as a therapeutic strategy for PCa is both an opportunity and a challenge, warranting further research. In the future, it will be necessary to conduct additional investigations of the interplay between the Wnt signaling pathway and ncRNAs and develop more reliable methods for targeting ncRNAs for their clinical application. Simultaneously, alternative therapeutic strategies for PCa should also be explored to improve the efficacy of treatment and the quality of life of patients.
